# Multi-Objective Optimization of Milling Process Parameters Using MOWOA and Comprehensive Performance Evaluation via AHP-TOPSIS

**DOI:** 10.3390/s26165212

**Published:** 2026-08-17

**Authors:** Fada Cai, Rongfei Xia

**Affiliations:** Chengyi College, Jimei University, Xiamen 361021, China

**Keywords:** multi-objective, orthogonal experiments, MOWOA, AHP, TOPSIS, material removal rate

## Abstract

To achieve the multi-objective collaborative optimization of milling processes, orthogonal experiments are conducted to develop prediction models for vibration acceleration and milling force, and range analysis together with variance analysis are adopted to reveal the sensitivity of each milling parameter to machining performance. Taking low vibration, small milling force and high material removal rate (MRR) as optimization objectives, the Multi-Objective Whale Optimization Algorithm (MOWOA) is employed to tackle this multi-criteria optimization problem, and a set of Pareto non-dominated solutions with balanced trade-offs are acquired. By integrating the weight assignment of the Analytic Hierarchy Process (AHP) with the Technique for Order Preference by Similarity to Ideal Solution (TOPSIS), comprehensive decision-making for all candidate schemes is implemented in accordance with practical machining requirements of users, and the optimal milling process parameters are determined. The results indicate an inherent trade-off among machining efficiency, milling load and machine tool vibration. An increase in the material removal rate will inevitably lead to simultaneous rises in milling force and machine tool vibration magnitude. The optimal combination of process parameters screened to meet comprehensive multi-objective requirements is spindle speed *n* = 12,000.00 r/min, feed rate *v*_f_ = 1048.26 mm/min, and axial milling depth *a*_p_ = 3.00 mm.

## 1. Introduction

Numerous practical engineering problems are characterized by conflicting performance objectives, among which the parameter optimization of milling processes stands as a typical representative [1,2,3]. Milling parameter design requires simultaneous trade-offs among machining quality, production cost and material removal efficiency. Higher milling parameters such as elevated feed rate and milling depth are generally adopted to boost productivity, yet they inevitably generate excessive milling forces and aggravate machine tool vibration, which deteriorates workpiece accuracy and the service life of cutters. Conventional single-objective optimization strategies can only achieve optimal performance for a single indicator and fail to balance multiple competing objectives. Accordingly, multi-objective optimization serves as a vital tool to coordinate contradictory milling parameters and achieve comprehensive balanced machining performance [4,5,6,7].

The core of multi-objective optimization (MOO) lies in identifying the optimal balance among multiple conflicting objectives, namely the so-called Pareto-optimal solutions. This method generates a set of alternative schemes, each of which achieves superior performance in certain indicators at the expense of compromises in others. It provides decision-makers with a wide range of feasible options, enabling them to make more rational decisions in accordance with practical requirements and objective priorities. In recent years, multi-objective optimization approaches have evolved into a vital research area across engineering disciplines [8,9,10]. Lahlali et al. [11] optimized the CNC milling process of 2017A aluminum alloy by jointly minimizing energy consumption and acoustic emission, two critical sustainability indicators. The NSGA-II algorithm was utilized for Pareto front generation, while TOPSIS was utilized for final parameter decision-making. The results revealed that the combination of high milling speed, low feed rate and large milling depth synchronously reduced energy consumption and acoustic emissions without sacrificing workpiece surface integrity. Pan et al. [12] implemented C60 nanofluid minimum quantity lubrication (NMQL) for the milling of Inconel 718 and developed a diversified mutation-driven multi-objective particle swarm optimization (MOPSO) algorithm to concurrently minimize milling force and residual stress while maximizing the material removal rate. Experimental validation verified that the C60-based NMQL reduced milling force by 24.92% and residual stress by 36.73%, compared with conventional minimum quantity lubrication (MQL). Such improvement demonstrates that the superior thermal and lubricative properties of nanofluids can effectively enhance workpiece dimensional stability and machining productivity. Natesh et al. [13] investigated the intrinsic trade-off between surface roughness and material removal rate during face milling of AISI 316L stainless steel. They screened critical milling parameters via the Taguchi method and constructed predictive models based on response surface methodology. Comparative analysis indicated that random forest regression (RFR) yielded the optimal prediction accuracy, which exhibits its strong robustness for capturing nonlinear machining correlations. Devi et al. [14] explored the milling performance of Custom 450 stainless steel with liquid-nitrogen cryogenically treated carbide tools. Targeting milling force, surface roughness and milling temperature as optimization metrics, they established machining prediction models using response surface methodology. A hybrid Taguchi–TOPSIS strategy was applied to optimize milling parameters, and the search performance of multiple algorithms was systematically compared. In terms of the inverted generational distance (IGD) convergence metric, the ant lion optimizer (ALO) delivered the best overall optimization capacity, proving its strong applicability to multi-objective parameter optimization for milling operations.

Compared with MOPSO and NSGA-II, MOWOA [15,16,17] integrates three collaborative search mechanisms, encircling prey, spiral update and random global search, which effectively alleviates premature convergence and local optimum trapping. The external archive preserves all non-dominated solutions generated during iterations, and the crowding degree strategy guides the population to evolve toward sparse regions of the Pareto front. Consequently, the proposed algorithm simultaneously guarantees the convergence accuracy and favorable distribution diversity of Pareto sets and can be widely applied to various engineering optimization problems.

Despite substantial research efforts devoted to parameter optimization for machining metallic materials, the milling process of wood remains relatively underexplored. In existing relevant studies, algorithmic performance evaluation often relies solely on qualitative visualization of the Pareto-optimal front. Furthermore, few optimization frameworks designed for wood machining have incorporated decision-maker preference information into the solution ranking process. To address the collaborative optimization problem of multiple milling parameters, a comprehensive optimization framework integrating orthogonal experiments [18,19], predictive modeling, MOWOA and hybrid AHP-TOPSIS decision-making is established. In conventional multi-objective optimization algorithms, objective functions are usually derived from empirical formulas. In stark contrast, the framework uniquely integrates orthogonal experiments with predictive modeling to initialize the heuristic search strictly within physically feasible parameter spaces, substantially curtailing invalid exploration compared to random initialization. This dedicated “experiment-to-algorithm” data-transfer protocol constitutes a distinct novel contribution to multi-objective milling optimization. Secondly, in the broader field of multi-objective optimization, most previous studies either terminate at acquiring the Pareto-optimal front or resort to straightforward weighted-sum approaches. This framework introduces a novel sequential screening rule: after MOWOA generates a dense Pareto set, TOPSIS is first applied to quantify the closeness of each solution to a virtual ideal solution, followed by AHP to resolve ties under conflicting preference criteria. This tailored “two-stage screening and ranking” architecture delivers engineering references that are substantially more reliable and physically interpretable than those derived from either method in isolation.

## 2. Materials and Methods

### 2.1. Materials

In the application scenario of CNC engraving-milling of wood, the dynamometer and accelerometer are adopted as conventional measurement instruments. A reliable experimental dataset is established through data acquisition to reflect the coupling relationships among process parameters, milling force and vibration response. For optimization modeling, vibration acceleration is identified as the primary optimization objective. This metric is critical for machining stability, yet it has not been sufficiently addressed in existing wood-milling investigations. This study demonstrates how signals from conventional sensors can be utilized to tackle an underexplored problem of practical engineering significance in the field of machining quality. Within this framework, the sensors serve as fundamental data-acquisition tools and provide crucial support for empirical modeling and subsequent multi-objective optimization.

**Figure 1:**
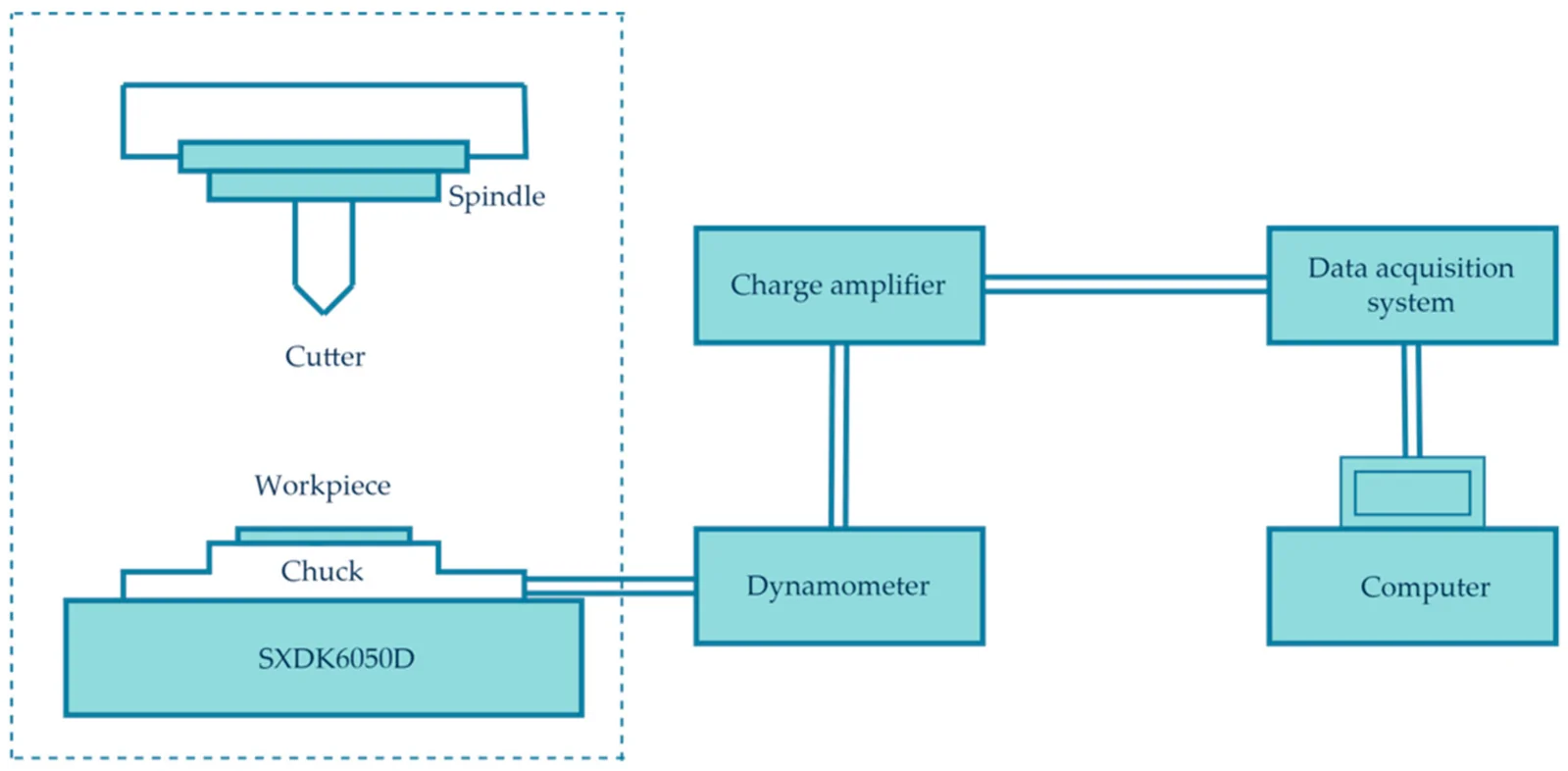
Schematic diagram of the milling force measurement system.

**Figure 2:**
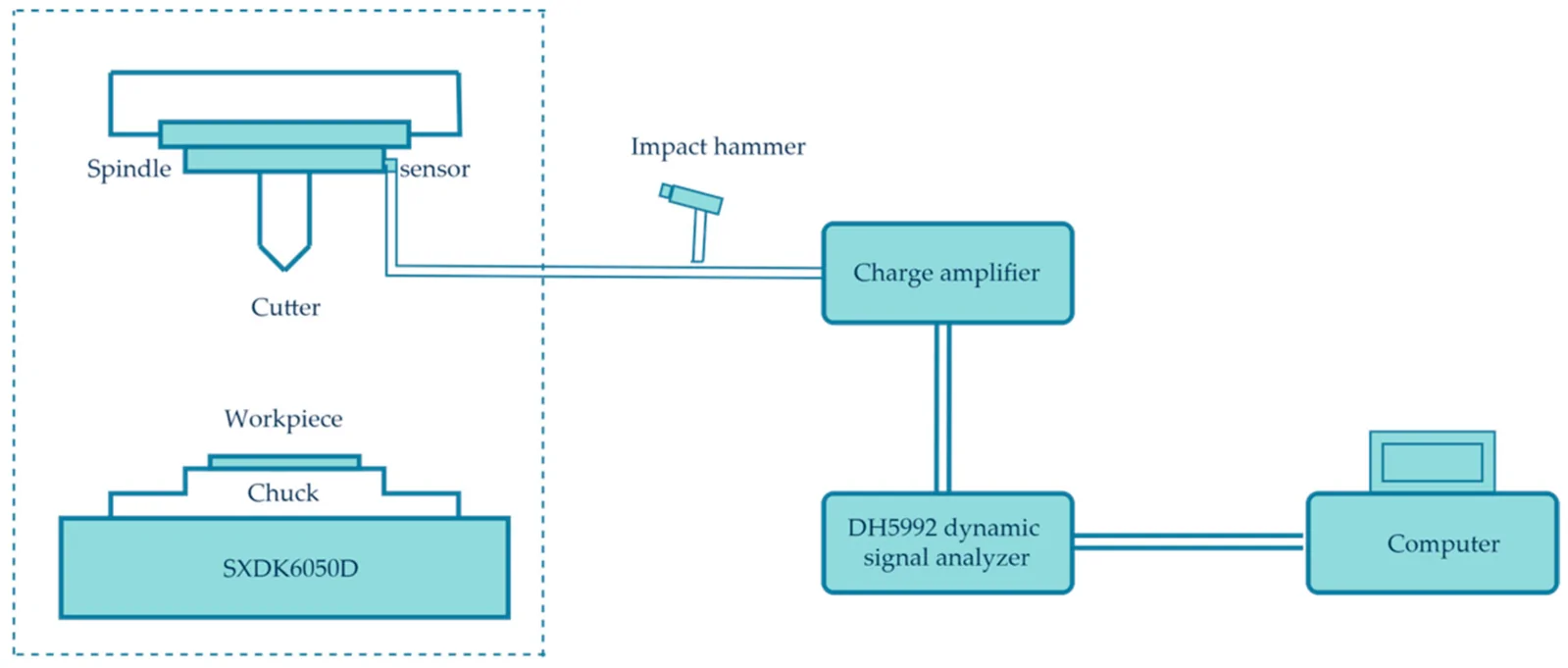
Schematic diagram of the vibration testing system.

The experiment was carried out on an SXDK6050D CNC engraving and milling machine (Taizhou Samsung Machine Tool Factory, Taizhou, China), featuring a maximum spindle speed of 24,000 r/min and a maximum feed rate of 6000 mm/min. Milling forces were measured using a Kistler 9257B three-dimensional dynamometer (Kistler Instrumente AG, Winterthur, Switzerland), interfaced with a Kistler 5070A1210 charge amplifier (Kistler Instrumente AG, Winterthur, Switzerland). Vibration responses were acquired via a PCB 356A0 piezoelectric accelerometer (PCB Piezotronics, Inc., Depew, NY, USA). Impact excitation was generated via an LNI-050 modal impact hammer (Jiangsu Lianneng Electronic Technology Co., Ltd., Yangzhou, China). A CA-YD-103 piezoelectric accelerometer from the same manufacturer was mounted on the surface of the spindle to capture vibration response signals, and the resulting dynamic signals were synchronously acquired and real-time processed by a DH5992 high-speed dynamic signal analyzer (Donghua Testing Technology Co., Ltd., Donghua, China). The configuration and signal transmission routes of the milling force measurement system and the machine tool vibration testing system are illustrated in Figure 1 and Figure 2, respectively.

The tool selected is a carbide double-edged straight-flute pointed cutter (Shanghai Micro-engraving CNC Machinery Co., Ltd., Shanghai, China), which features high hardness, excellent wear resistance, and impact toughness, making it suitable for stable wood processing under medium-to-high-speed cutting conditions [20,21]. Its symmetrical double-edge design helps to balance cutting forces, reducing vibration during machining, thereby improving surface quality and dimensional stability. The workpiece material is naturally dried rosewood (Guangzhou Jiabao Mahogany Furniture Co., Ltd., Guangdong, China), known for its uniform structure and widely used in precision woodworking products [22,23]. The performance parameters of cutters and workpieces are detailed in Table 1 and Table 2.

### 2.2. Methods

**Figure 3:**
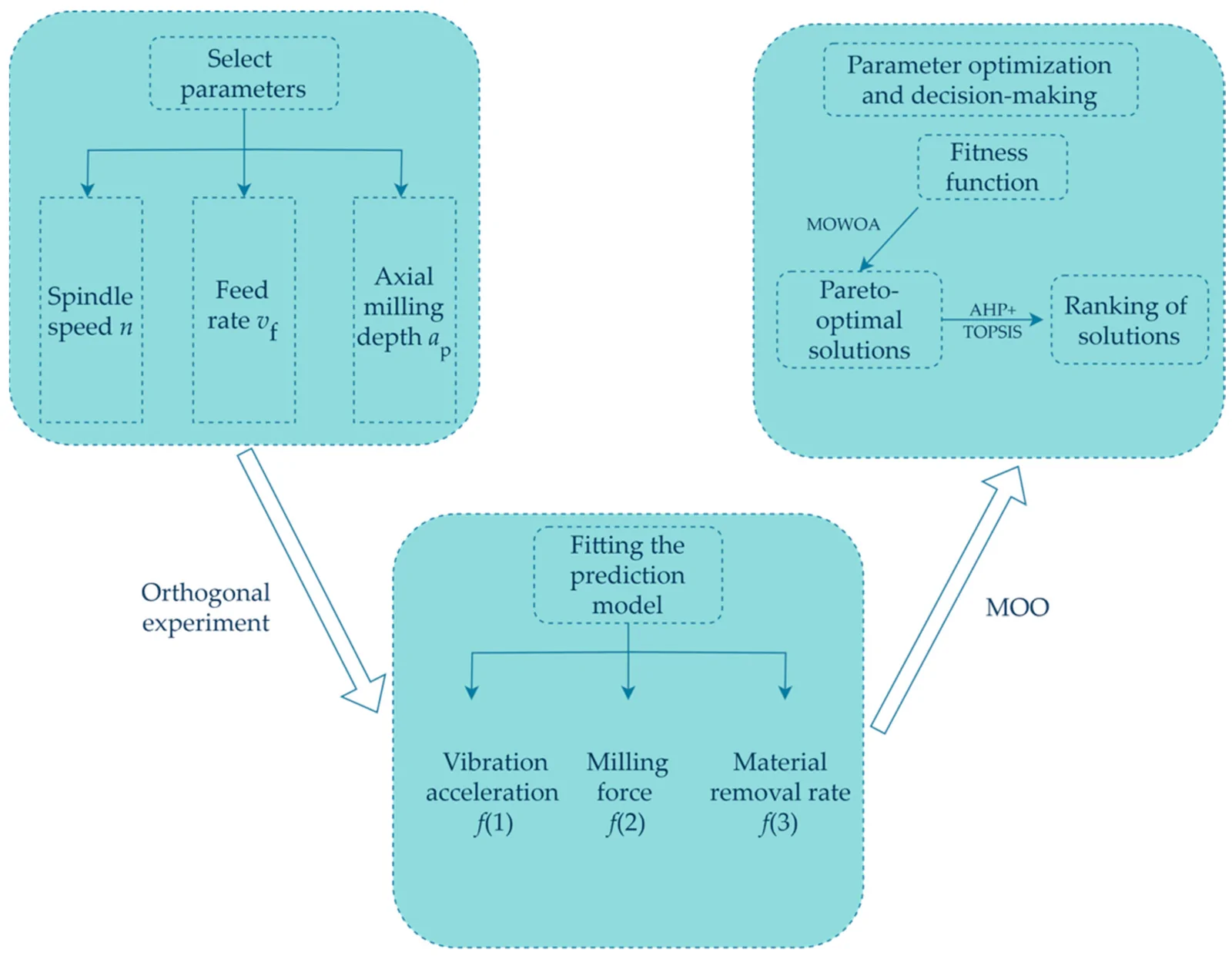
Overall experimental framework.

The overall experimental framework consists of three sequential, interdependent stages: (1) parameter screening by orthogonal experimental design, which implements a three-factor three-level scheme as listed in Table 3; (2) construction and validation of the prediction model; (3) multi-objective optimization coupled with decision support analysis. As illustrated in Figure 3, the process begins with an orthogonal experimental design to systematically explore the parameter space, identify influential factors, and efficiently collect high-quality experimental data—thereby enabling robust fitting of the target prediction model. Subsequently, a multi-objective fitness function is formulated based on the validated prediction model, and its Pareto-optimal solution set is obtained using the MOWOA, ensuring global search capability and convergence reliability. Finally, objective weights are rigorously derived through the AHP, and the non-dominated solutions are prioritized with the aid of the TOPSIS, yielding a final optimal solution for practical decision-making.

#### 2.2.1. Experimental Models

During machining operations, machine tool vibration constitutes a critical factor influencing machining quality, dimensional accuracy, and tool wear. The corresponding vibration acceleration model is formulated as an exponential empirical equation [24]:

(1) $$a=C\cdot n^{\gamma_{1}}\cdot v_{f}^{\gamma_{2}}\cdot a_{p}^{\gamma_{3}}$$

where *a* denotes the machine tool vibration acceleration; *C* is the machining coefficient—a dimensionless empirical parameter whose value depends on multiple interrelated factors, including workpiece material, cutting tool geometry, and specific machining conditions. The variables *n*, *v*_f_, and *a*_p_ represent the spindle speed, feed rate, and axial milling depth, respectively. The exponents *γ*_1_, *γ*_2_, and *γ*_3_ are empirically determined power-law exponents associated with *n*, *v*_f_, and *a*_p_, respectively, reflecting their nonlinear contributions to vibration acceleration.

The milling force *F* is modeled using a quadratic regression polynomial as a function of spindle speed *n*, feed rate *v*_f_, and axial milling depth *a*_p_ [24]:

(2) $$F=\beta_{0}+\beta_{1}n+\beta_{2}v_{f}+\beta_{3}a_{p}+\beta_{12}nv_{f}+\beta_{13}na_{p}+\beta_{23}v_{f}a_{p}+\beta_{11}n^{2}+\beta_{22}v_{f}^{2}+\beta_{33}a_{p}^{2}$$

where *β*_0_, *β*_1_, …, *β*_33_ denote the undetermined polynomial regression coefficients.

In practical engineering applications, multiple objectives commonly exhibit interdependence and intrinsic conflict. In contrast to single-objective optimization, multi-objective optimization explicitly incorporates the trade-offs and frequently irreconcilable conflicts among competing objectives to determine Pareto-optimal solutions. Its mathematical formulation is given by:

(3) $$\left\{\begin{array}{l} min\;Y\left(x\right)={\left[y_{1}\left(x\right),y_{2}\left(x\right),\cdots ,y_{n}\left(x\right)\right]}^{T}\\ s.t\;x\in \Omega \\ \Omega =\left\{x\in R^{m}\left|g_{i}\left(x\right)\leq 0,i=1,2,\cdots ,J\right.\right\} \end{array}\right.$$

where ***Y***(**x**) denotes the multi-objective function vector with a dimension of *n*; *y_i_*(***x***) represents the *i*-th single objective function. ***x*** = [*x*_1_, *x*_2_, …, *x*_m_] stands for the decision variable vector within the decision space Ω; *g_i_*(***x***) indicates the *i*-th inequality constraint; and *J* is the total number of inequality constraints.

#### 2.2.2. Experimental Algorithms

1. Multi-Objective Whale Optimization Algorithm

**Figure 4:**
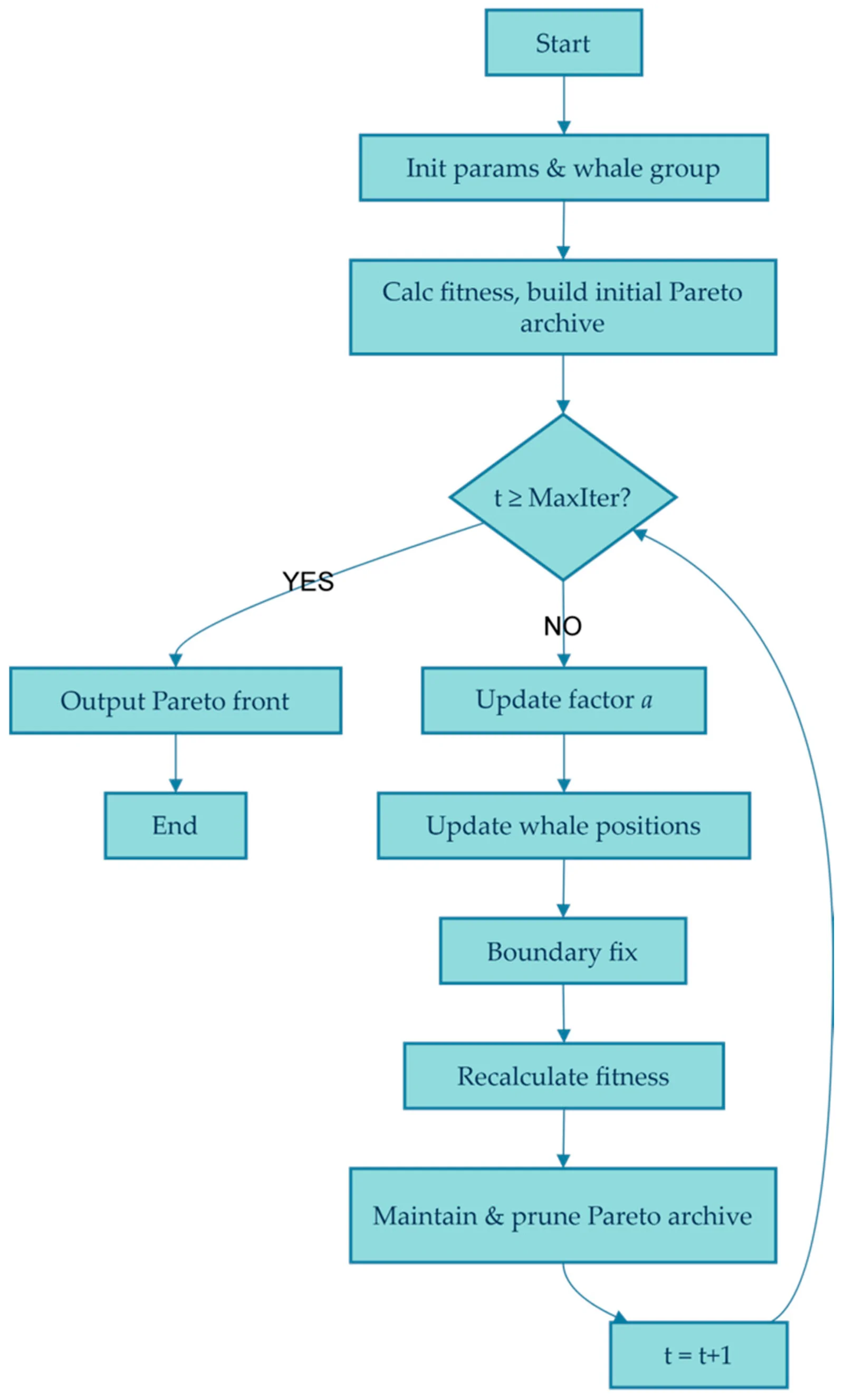
The flowchart of MOWOA.

The execution procedure of MOWOA is illustrated in Figure 4, with its core iterative steps summarized as follows: (1) Randomly initialize the whale population. (2) Evaluate the multi-objective fitness values of all individuals. (3) Screen non-dominated solutions from the initial population through fast non-dominated sorting and store them in the external archive. (4) Termination judgment: If the stopping criterion is satisfied, the converged Pareto-optimal solution set is extracted from the external archive. Otherwise, the algorithm updates the convergence parameter *a*, renews whale positions via the bubble-net foraging exploitation or global exploration strategy, and corrects out-of-bounds individuals with boundary constraints. After recalculating the multi-objective fitness of updated individuals, the external archive is updated and pruned. The iteration counter is then incremented by one, and the algorithm jumps back to the termination judgment step to check the stopping criterion again.

2. AHP and TOPSIS Algorithms

**Figure 5:**
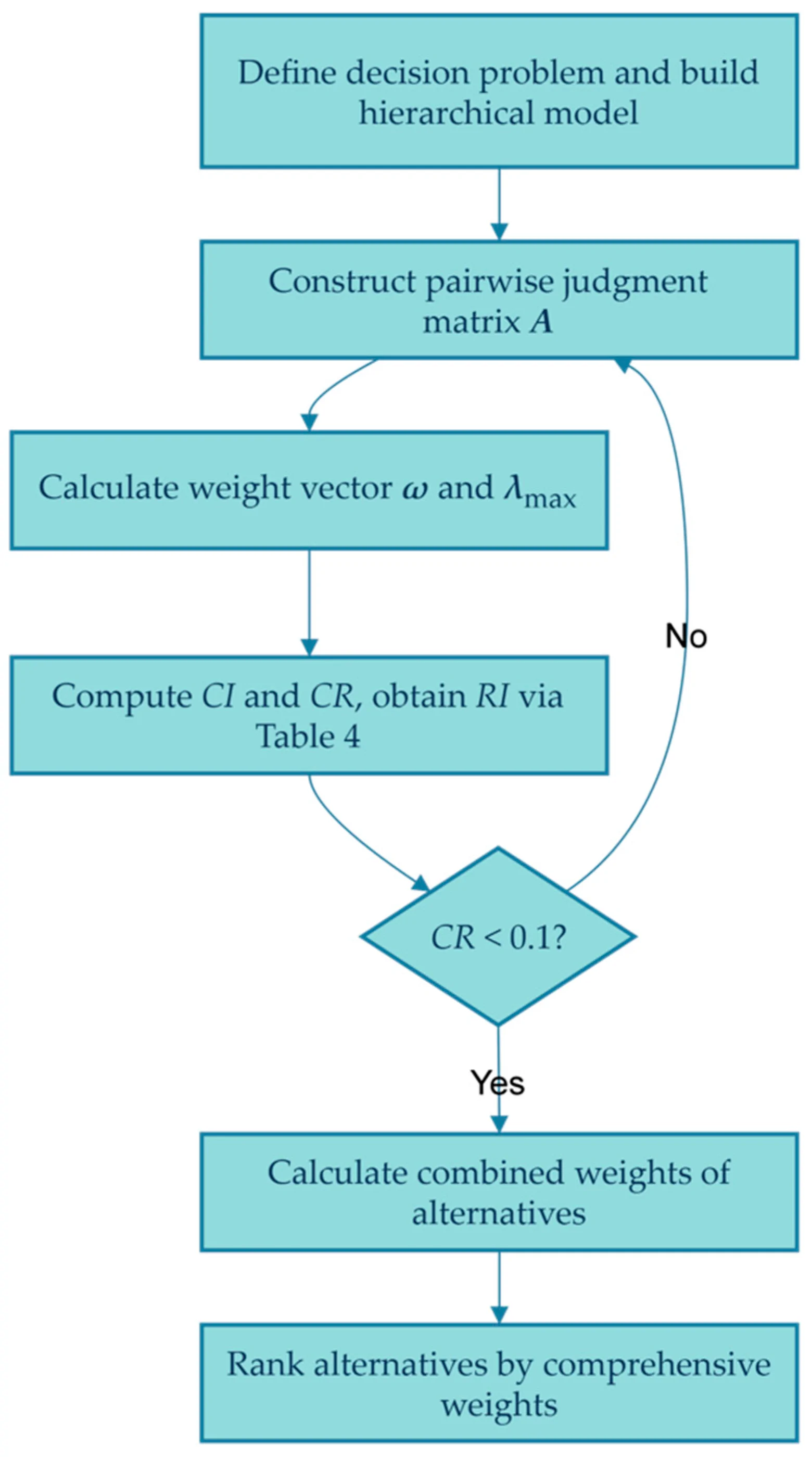
The flowchart of AHP.

AHP decomposes decision-making elements into multiple hierarchical layers consisting of objectives and criteria to construct a hierarchical structural model. It quantifies the relative importance of each element via pairwise comparisons and further calculates the weight coefficient corresponding to each objective. The corresponding calculation flowchart is presented in Figure 5, which can be summarized as follows: (1) Clarify the decision-making problem and corresponding objectives. (2) Establish the judgment matrix ***A*** = [*a_ij_*]*_m_*_×*m*_, where *a_ij_* denotes the relative importance of criterion *i* with respect to criterion *j*, and *m* stands for the dimension of the judgment matrix. (3) Solve for the weight vector ***ω***, and compute the maximum eigenvalue *λ*_max_ as well as the corresponding eigenvector ***w*** of the judgment matrix. (4) Carry out a consistency test by calculating the consistency index *CI* and consistency ratio *CR* [25].

(4) $$CI=\frac{\lambda_{max}-m}{m-1}$$

(5) $$CR=\frac{CI}{RI}$$

where the value of *RI* can be obtained by looking it up in Table 4 according to the order *m* of the judgment matrix. If *CR* < 0.1, the judgment matrix is considered to have satisfactory consistency; otherwise, the judgment matrix needs to be readjusted.

**Figure 6:**
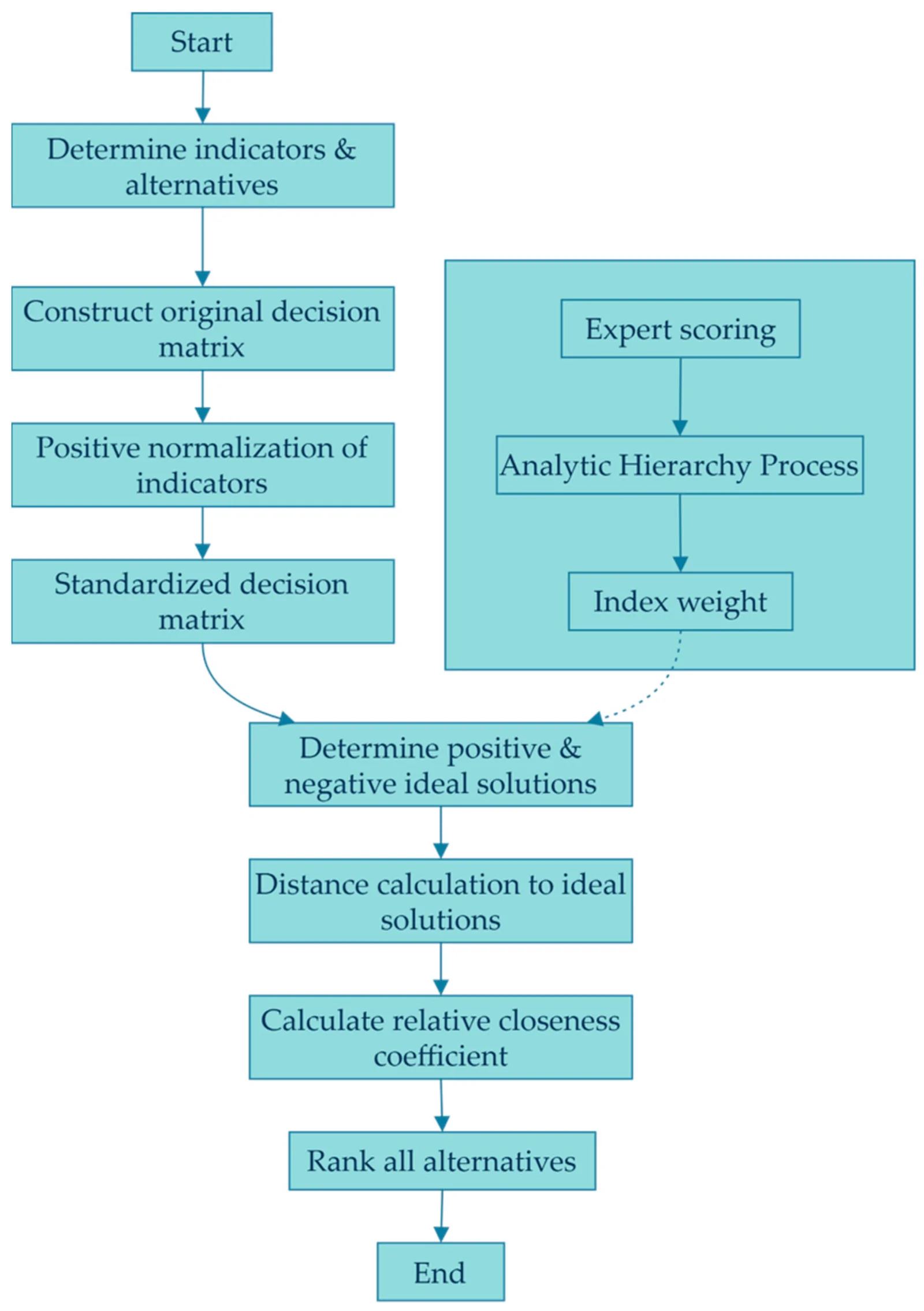
The flowchart of TOPSIS.

TOPSIS ranks alternatives under multiple criteria by evaluating their relative closeness to the positive and negative ideal solutions while integrating the weight information of each criterion, so as to support scientific and rational decision-making. Its detailed implementation steps are presented in Figure 6.

Given *n* alternatives and *m* indicators, the standardized decision matrix is ***Z*** = [z*_ij_*]*_n_*_×*m*_. The positive ideal solution *z_j_*^+^ and negative ideal solution *z_j_*^−^ for each indicator are defined below:

(6) $$\left\{\begin{array}{l} z_{j}^{+}=max\left(z_{1j},\;z_{2j},\;\cdots ,\;z_{mj}\right)\\ z_{j}^{-}=min\left(z_{1j},\;z_{2j},\;\cdots ,\;z_{mj}\right) \end{array}\right.$$

Then the weighted Euclidean distances from the *i*-th evaluation alternative to the positive and negative ideal solutions are denoted as *D_i_*^+^ and *D_i_*^−^, respectively [26].

(7) $$\left\{\begin{array}{l} D_{i}^{+}=\sqrt{\sum_{j=1}^{m}\omega_{j}{\left(z_{j}^{+}-z_{ij}\right)}^{2}}\\ D_{i}^{-}=\sqrt{\sum_{j=1}^{m}\omega_{j}{\left(z_{j}^{-}-z_{ij}\right)}^{2}} \end{array}\right.$$

where *ω_j_* represents the weight coefficient of the *j*-th evaluation indicator. Then the evaluation score of the *i*-th alternative, denoted as *S_i_*, can be expressed as follows [26].

(8) $$S_{i}=\frac{D_{i}^{-}}{D_{i}^{-}+D_{i}^{+}}$$

## 3. Results

### 3.1. Results of Orthogonal Experiments

Three factors, namely spindle speed, feed rate and axial milling depth, each with three levels, were selected in the experiment. The orthogonal array *L*_18_ (3^7^) was adopted to conduct the milling tests. The measured orthogonal test results of machine tool vibration acceleration *a* and milling force *F* under various combinations of milling parameters are listed in Table 5.

Range analysis (RA) and analysis of variance (ANOVA) [27,28] were performed on the measured machine tool vibration acceleration in Table 5, and the corresponding results are summarized in Table 6 and Table 7. Comparison of range values yields the order *n* (2.813) > *a*_p_ (1.413) > *v*_f_ (1.026), which indicates that the descending order of the influence of milling parameters on vibration acceleration is spindle speed *n*, axial milling depth *a*_p_, and feed rate *v*_f_. With the significance level set to *α* = 0.05, the *p*-values obtained from ANOVA follow *n* (0.002) < *a*_p_ (0.0147)< *v*_f_ (0.034) < *p*-values (0.05), verifying that spindle speed, axial milling depth and feed speed all exhibit significant correlations with vibration acceleration. From the perspective of cutting dynamics, an increase in spindle speed raises the centrifugal force of the rotary cutter and the excitation induced by the tooth passing impact, thereby aggravating machine tool vibration. Meanwhile, larger axial milling depth and feed rate increase the instantaneous cutting load, which further amplifies the vibration response of the whole machine tool.

For the milling force, RA and ANOVA, as listed in Table 8 and Table 9 separately, reveal that the range values satisfy *a*_p_ (11.805) > *v*_f_ (8.122) > *n* (2.077). The priority order of parameter influence on milling force is axial milling depth *a*_p_, feed rate *v*_f_, and spindle speed *n*. The ranking of ANOVA *p*-values is *a*_p_ (6.98 × 10^−5^) < *v*_f_ (0.0010) < *p*-values (0.05), demonstrating that milling force is significantly correlated with axial milling depth and feed rate. In terms of cutting mechanism, the growth of axial milling depth deepens the cutter engagement depth, which elevates the shear stress generated during the milling process. In addition, a higher feed rate extends the cutting distance and material removal volume per unit time, requiring a larger cutting force to overcome the resistance caused by plastic deformation of the workpiece material.

Table 10 lists the significance of interaction and quadratic terms in the quadratic response surface model. Among the cross-product interaction terms, only *n*∙*a*_p_ exhibits statistical significance, indicating that the coupling effect between spindle speed *n* and axial milling depth *a*_p_ cannot be ignored for milling-force response. By contrast, the interaction *n*∙*v*_f_ and *v*_f_∙*a*_p_ are non-significant. In addition, all three quadratic terms show no obvious statistical influence on milling force. It reveals that the nonlinear quadratic self-effect of individual process parameters is weak within the investigated parameter range.

### 3.2. Validation of the Prediction Model

**Figure 7:**
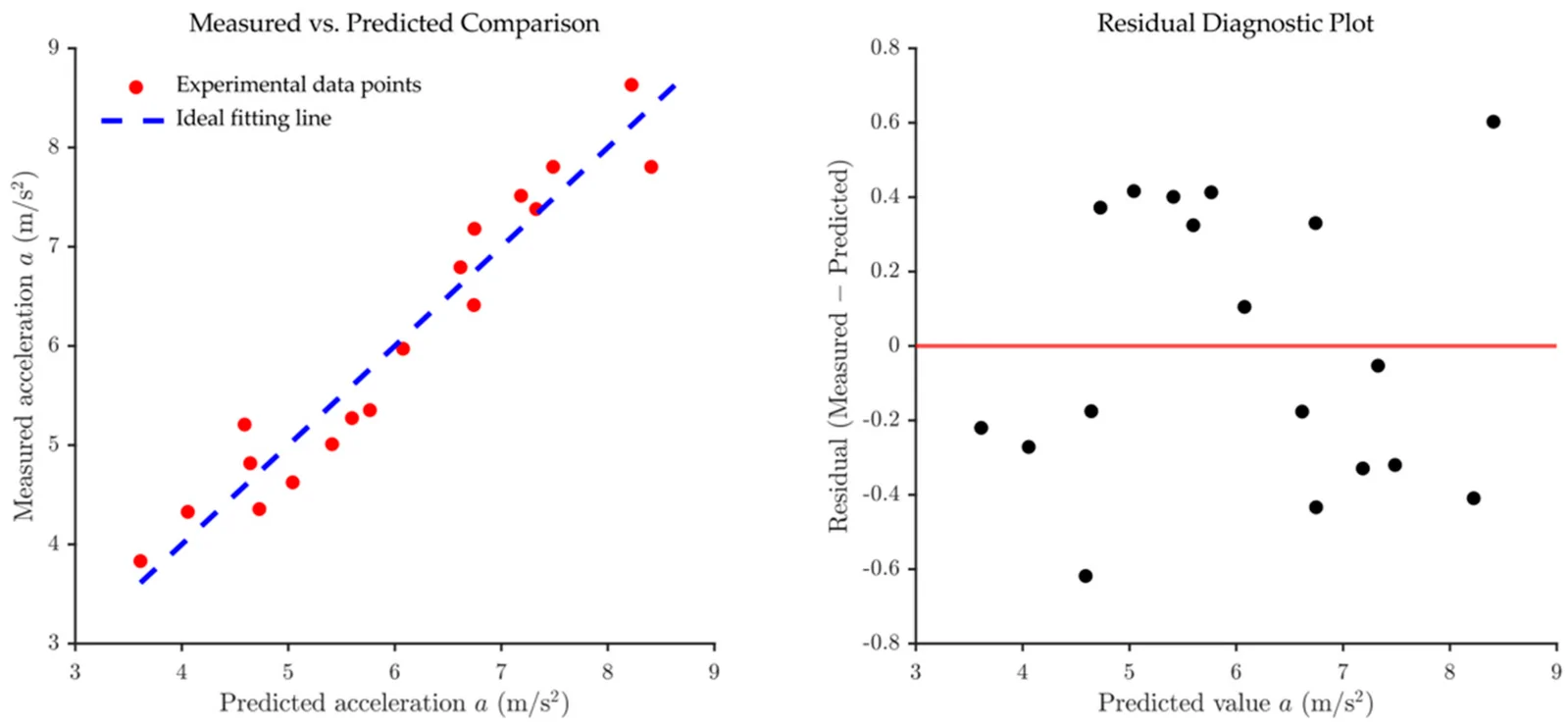
Vibration acceleration fitting diagnostic plots.

Equation (1) was implemented to fit the experimental data of vibration acceleration *a* provided in Table 5. The fitting results are presented in Table 11 and Table 12, while the diagnostic plots for the fitted models are illustrated in Figure 7. As shown in the tables, none of the 95% confidence intervals for the coefficients contain zero. The narrow interval widths demonstrate high statistical reliability of the parameter estimates. The coefficient of determination *R*^2^ reaches 0.934. As an indicator quantifying how well independent variables predict the dependent variable, *R*^2^ approaches 1 for higher model prediction accuracy [29]. Evaluated via leave-one-out cross-validation (LOOCV), the model yields a mean absolute percentage error (MAPE) of 7.48% and a root-mean-square error (RMSE) of 0.4807. The MAPE difference between full-data fitting and cross-validation is less than 2%, revealing that the empirical formula possesses strong generalization ability with no obvious overfitting. Therefore, the formula can be reliably adopted to predict vibration acceleration under the given machining conditions. As illustrated in the measured–predicted comparison and residual diagnostic plots, the vast majority of red experimental data points lie closely along the ideal blue fitting line. Residual points are randomly scattered on both sides of the zero-reference red line without a consistent unilateral bias. The above features demonstrate that the predicted values of the model are highly consistent with the variation trend of measured data, and no systematic bias exists, which verifies the reliable overall prediction performance of the model.

After the fitted parameters are substituted into Equation (1), the machine tool vibration acceleration *a* is formulated as:

(9) $$a=3.572\times 10^{-6}n^{1.207}\cdot v_{f}^{0.286}\cdot a_{p}^{0.469}$$

From the equation, the power exponents *γ*_1_, *γ*_2_, *γ*_3_ for spindle speed, feed rate and axial milling depth are positive with *γ*_1_ > *γ*_3_ > *γ*_2_. Thus, spindle speed dominates the vibration acceleration, axial milling depth ranks second, and feed rate has the least impact, which is consistent with the range analysis in Table 6.

**Figure 8:**
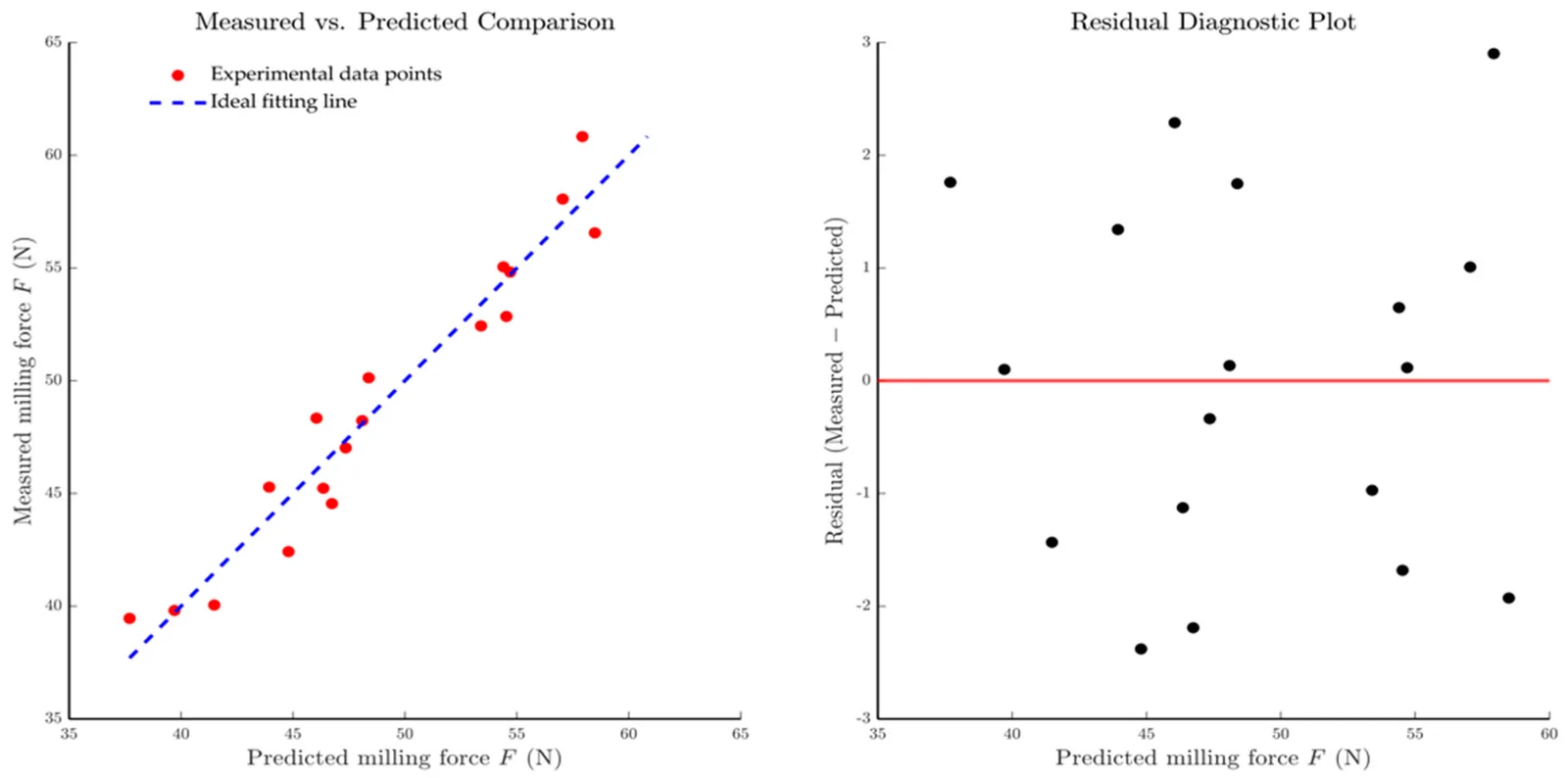
Milling force fitting diagnostic plots.

Equation (2) was applied to model the experimentally measured milling force *F* given in Table 5. The fitting results for the quadratic response surface model are presented in Table 13 and Table 14, and the corresponding diagnostic plots are illustrated in Figure 8. All regression coefficients are reported with their 95% confidence intervals. Owing to the limited degrees of freedom (df = 8), certain confidence intervals are relatively broad, indicating considerable uncertainty in individual coefficient estimates. However, this does not diminish the model’s overall predictive power, as evidenced by its excellent explanatory ability (*R*^2^ = 0.939) and satisfactory generalization performance under leave-one-out cross-validation (LOOCV MAPE = 6.55%). A comparison between calibration and cross-validation reveals a MAPE increase of approximately 3.8 percentage points, suggesting a slight overfitting tendency. Nonetheless, the cross-validated MAPE remains substantially below the conventional engineering tolerance of 10%, demonstrating that the developed RSM model is a reliable and practical tool for predicting milling force and analyzing process trends under the given conditions. As observed in Figure 8, all red experimental sample points are closely distributed along the ideal blue fitting line. When the predicted milling force ranges from 38 N to 61 N, the measured milling force increases linearly synchronously, showing a highly consistent variation trend between measured points and the idea line. Residuals scatter randomly and evenly on both sides of the zero-residual baseline, with roughly equal amounts of positive and negative errors. The above results verify that the fitted model can accurately characterize the variation law of milling force and possesses outstanding fitting precision and prediction reliability.

Substitution of the fitted parameters from Table 13 into Equation (2) yields the prediction formula of milling force:

(10) $$\begin{array}{l} F=70.821-6.100\times 10^{-3}n+0.012v_{f}-3.298a_{p}-5.301\times 10^{-7}nv_{f}+8.395\times 10^{-4}na_{p}\\ \;\;\;\;\;-2.763\times 10^{-4}v_{f}a_{p}+1.275\times 10^{-7}n^{2}+2.135\times 10^{-6}v_{f}^{2}-0.397a_{p}^{2} \end{array}$$

### 3.3. Multi-Objective Optimization Results and Trade-Off Analysis Among Conflicting Objectives

Machine tool vibration, milling force and material removal efficiency present a complex coupled relationship with mutual restrictions. In practical machining processes, increasing feed rate or axial milling depth is commonly utilized to improve milling efficiency. Nevertheless, such process adjustments simultaneously aggravate milling load and amplify machine tool vibration. Accordingly, exploring the dynamic trade-off law among the three indicators is of great significance for optimizing process parameters to balance machining quality and production efficiency.

The fitness functions of machine tool vibration acceleration and milling force are given as follows:

(11) $$\left\{\begin{array}{l} a=3.572\times 10^{-6}n^{1.207}v_{f}^{0.286}a_{p}^{0.469}\\ F=70.821-6.100\times 10^{-3}n+0.012v_{f}-3.298a_{p}-5.301\times 10^{-7}nv_{f}+8.395\times 10^{-4}na_{p}\\ \;\;\;\;\;-2.763\times 10^{-4}v_{f}a_{p}+1.275\times 10^{-7}n^{2}+2.135\times 10^{-6}v_{f}^{2}-0.397a_{p}^{2} \end{array}\right.$$

The material removal rate *Q* can be expressed as [30]:

(12) $$Q=\frac{v_{f}\cdot a_{p}\cdot a_{e}}{1000}$$

where *a*_e_ denotes the radial milling depth. An excessively large radial milling depth will deteriorate the machined surface quality, while an overly small value reduces milling efficiency. Based on practical machining experience, *a*_e_ is fixed at 1 mm in this study.

Taken together, the fitness functions for multi-objective optimization derived above are presented as follows:

(13) $$\left\{\begin{array}{l} \mathrm{Min}\;f\left(1\right)=3.572\times 10^{-6}n^{1.207}v_{f}^{0.286}a_{p}^{0.469}\\ \mathrm{Min}\;f\left(2\right)=70.821-6.100\times 10^{-3}n+0.012v_{f}-3.298a_{p}-5.301\times 10^{-7}nv_{f}+8.395\times 10^{-4}na_{p}\\ \;\;\;\;\;-2.763\times 10^{-4}v_{f}a_{p}+1.275\times 10^{-7}n^{2}+2.135\times 10^{-6}v_{f}^{2}-0.397a_{p}^{2}\\ \mathrm{Min}\;f\left(3\right)=-\frac{v_{f}\cdot a_{p}\cdot a_{e}}{1000} \end{array}\right.$$

The optimization constraints are specified as follows:

(14) $$\left\{\begin{array}{l} 12000\leq n\leq 18000\\ 1000\leq v_{f}\leq 2000\\ 3\leq a_{p}\leq 5 \end{array}\right.$$

The algorithm parameters were set as follows: a population size of 50, a maximum iteration number of 300, and an external archive capacity of 150. To assess the robustness of the stochastic MOWOA algorithm, 30 independent runs were conducted. The population size and iteration count follow standard MOWOA configurations; the archive size is three times the population size to preserve solution diversity; and 30 independent runs are conducted to ensure statistical reliability of the results. Table 15 summarizes the statistical results of the hypervolume (HV) indicator and the number of Pareto solutions obtained across these runs. The HV indicator exhibits a mean value of 0.0119 with an extremely low standard deviation of 0.0001, corresponding to a coefficient of variation (CV) of only 1.15%. This remarkably low CV demonstrates that MOWOA consistently converges to high-quality Pareto fronts with minimal variability across different random seeds. Furthermore, the number of Pareto solutions consistently reaches the archive capacity of 150 in every run, with zero standard deviation, indicating that the algorithm reliably generates a sufficient and stable set of non-dominated solutions. These results collectively confirm that MOWOA possesses strong robustness and stability for the present multi-objective optimization problem, despite its inherent stochastic nature.

**Figure 9:**
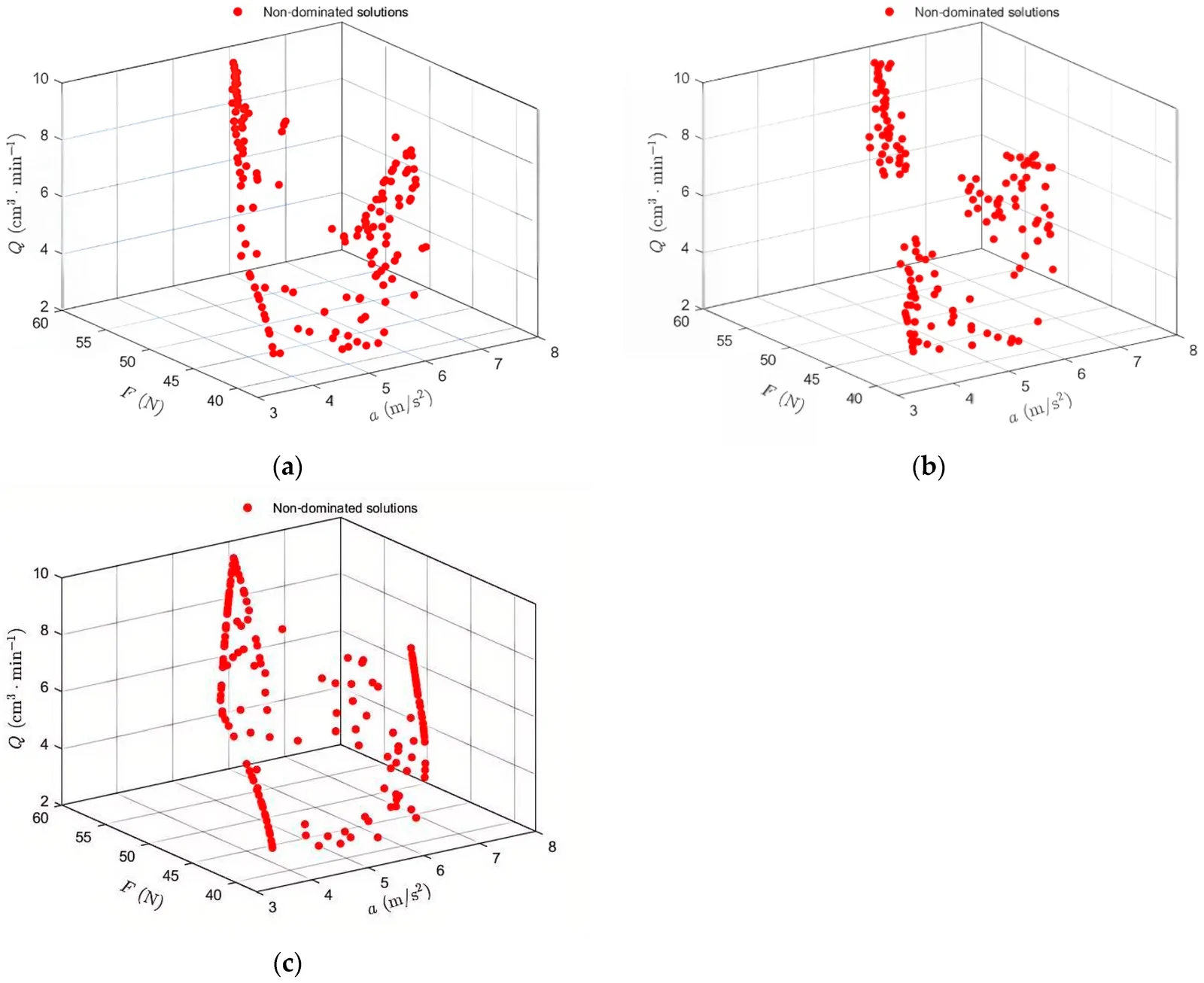
Comparison of Pareto-optimal fronts obtained by MOWOA, NSGA-II and MOPSO. (**a**) Pareto fronts of MOWOA. (**b**) Pareto fronts of NSGA-II. (**c**) Pareto fronts of MOPSO.

The three-dimensional Pareto-optimal fronts obtained by MOWOA, NSGA-II and MOPSO are compared in Figure 9. As illustrated in Figure 9, MOWOA yields a distinctly denser and more evenly distributed Pareto front than NSGA-II and MOPSO. In contrast, the Pareto fronts of NSGA-II and MOPSO exhibit notable gaps and localized clustering, which typically indicate premature convergence to sub-optimal regions—a common symptom of local optima entrapment in multi-modal objective spaces. To quantitatively substantiate this visual observation, the HV indicator—the most widely accepted unary performance metric in multi-objective optimization, as it simultaneously evaluates both convergence and diversity—was employed. The proposed MOWOA achieves the highest HV value (0.0119), surpassing NSGA-II (0.01123) by 5.97% and MOPSO (0.01104) by 7.79%. This confirms that MOWOA consistently generates Pareto fronts with superior overall quality.

**Figure 10:**
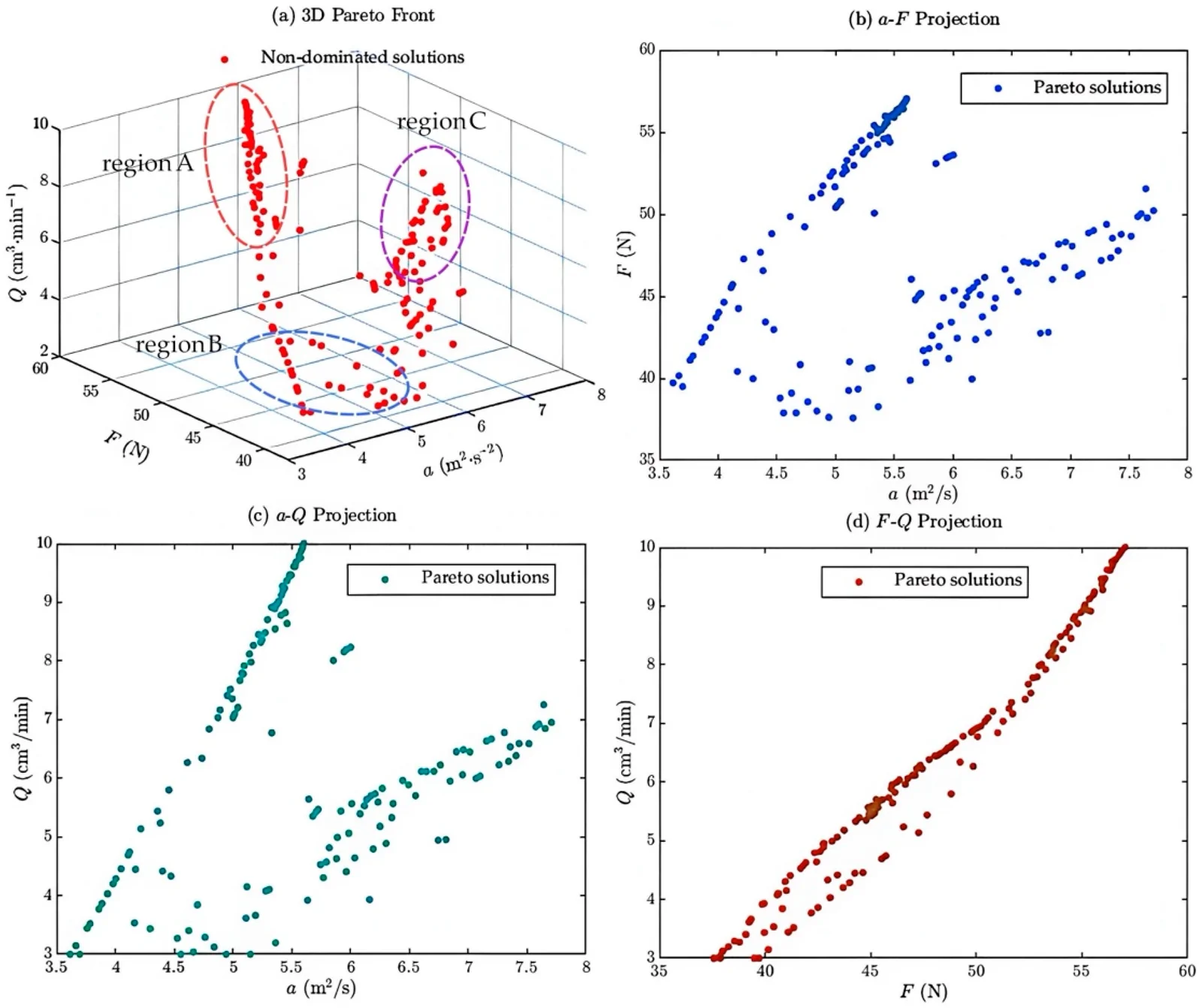
Pareto fronts of vibration acceleration *a*, milling force *F*, and material removal rate *Q* and two-dimensional projections.

The Pareto fronts of vibration acceleration *a*, milling force *F*, and material removal rate *Q*, together with their two-dimensional projections, were finally obtained, as shown in Figure 10. In Figure 10a, the non-dominated optimal solutions are uniformly distributed in a continuous inclined strip without missing intervals, which reflects the superior convergence performance of the algorithm. The majority of the Pareto frontier can be divided into three distinct trade-off regions: (i) the high-efficiency region (region A), associated with elevated milling forces and moderate vibration; (ii) the low-vibration region (region B), exhibiting reduced milling forces but the lowest material removal rate; (iii) the high-vibration region (region C), wherein machining efficiency and milling forces are both moderate. Figure 10b reveals a monotonic positive correlation between milling force and vibration acceleration. Combined with Figure 10c,d, an inherent trade-off exists between machining efficiency, milling load and vibration. Increasing the material removal rate will inevitably aggravate the milling force and machine tool vibration.

In the experiment, judgment matrix ***A*** is introduced for the three evaluation indices: machine tool vibration acceleration *a*, milling force *F*, and material removal rate *Q*.

(15) $$A=\left[\begin{array}{ccc} 1 & 4 & 2\\ 1/4 & 1 & 1/2\\ 1/2 & 2 & 1 \end{array}\right]$$

The maximum eigenvalue of the judgment matrix is solved as *λ*_max_ = 3, with a consistency index *CI* = 0 and a consistency ratio *CR* = 0 < 0.1, which means the matrix passes the consistency test. The corresponding indicator weight vector is ***ω*** = (0.571, 0.143, 0.286)^T^, indicating that the weight proportions of the three evaluation indicators, machine tool vibration acceleration *a*, milling force *F* and material removal rate *Q* are 57.1%, 14.3% and 28.6%, respectively.

The sensitivity analysis in Table 16 indicates that the optimal solution shifts from productivity-oriented toward quality-oriented as the weight of vibration acceleration increases. The sensitivity analysis demonstrates that the baseline AHP weights (***ω*_a_** = 0.571) yield an optimal solution that is fully consistent with the engineering priority of vibration suppression. While alternative weight configurations select different Pareto-optimal solutions—favoring either productivity (*ω*_a_ ≤ 0.571) or quality (*ω*_a_ ≥ 0.571)—the baseline solution maintains a balanced trade-off between the three objectives. The observed transition near *ω*_a_ ≈ 0.54 indicates a natural trade-off region on the Pareto front, confirming the validity of the optimization results and the rationality of the AHP-based decision-making framework.

According to the actual machining evaluation requirements, machine tool vibration acceleration *a* and milling force *F* are classified as cost-type indicators, where smaller values correspond to better machining conditions; the material removal rate *Q* is defined as a benefit-type indicator, and a larger value denotes higher machining efficiency. AHP and TOPSIS are combined to conduct a comprehensive performance ranking of all 150 groups of milling process parameter combinations. A higher comprehensive closeness score implies that the corresponding process scheme better satisfies practical machining demands.

**Figure 11:**
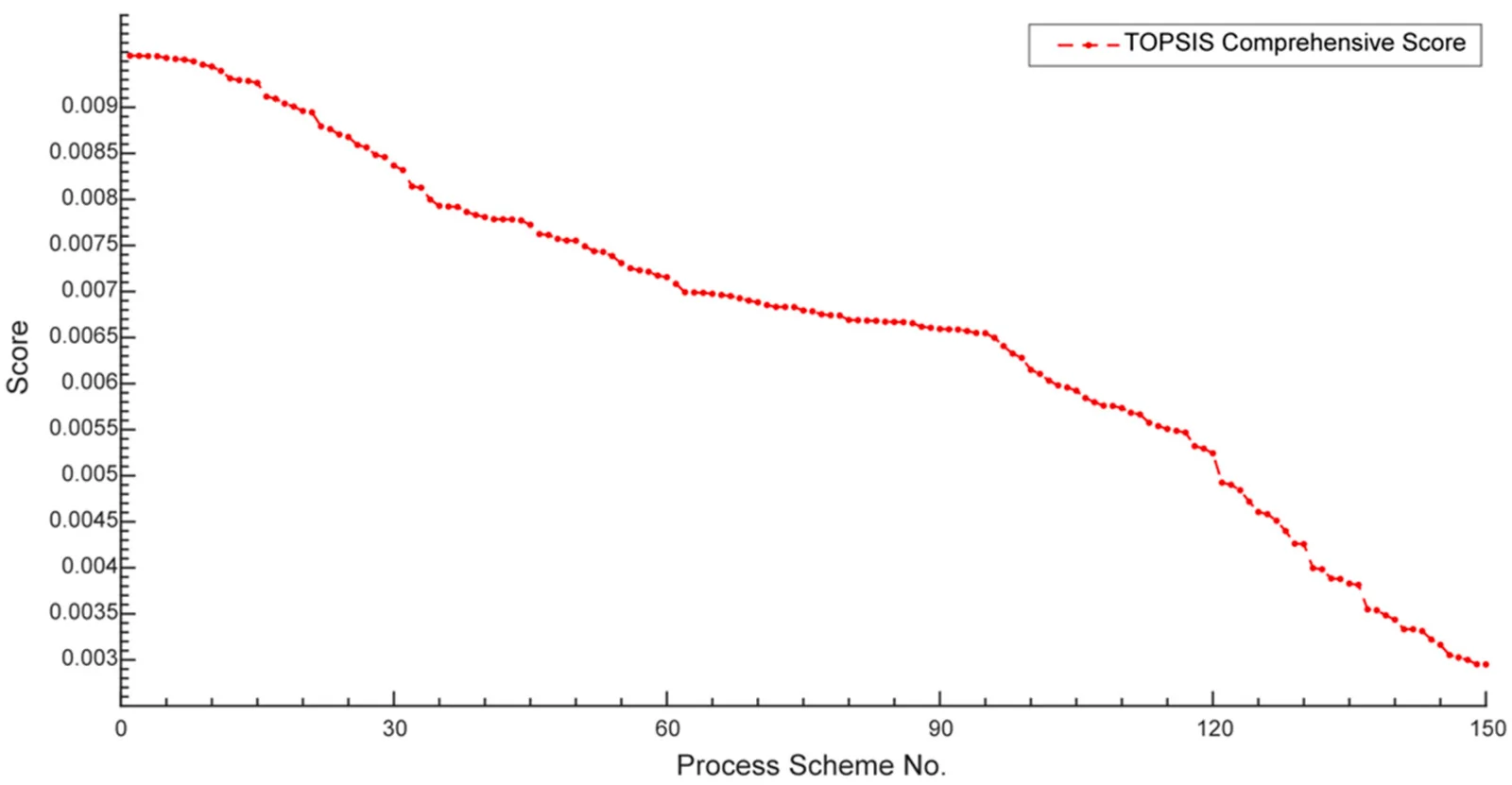
Descending order of TOPSIS scores for milling processes.

Figure 11 presents the comprehensive evaluation results of all parameter schemes, and Table 17 summarizes the top 30 optimal process combinations in terms of comprehensive performance. It can be concluded from the table that the optimal milling process parameters that meet the evaluation objectives are spindle speed *n* = 12000.00 r/min, feed rate *v*_f_ = 1048.26 mm/min, and axial milling depth *a*_p_ = 3.00 mm.

To validate the predictive reliability of the established empirical models, the predicted values were compared with experimental measurements under both conventional and optimal parameter settings, as presented in Table 18. The prediction errors for vibration acceleration and milling force are within 6% for both parameter sets, confirming that the developed models exhibit satisfactory accuracy and practical applicability for engineering purposes. Under the optimal parameter set, the measured vibration acceleration decreases by 46.00% (from 6.421 m/s^2^ to 3.467 m/s^2^) and the milling force decreases by 21.99% (from 49.543 N to 38.642 N), compared with the conventional settings. This is achieved at the cost of a 47.58% reduction in material removal rate (from 6.000 cm^3^/min to 3.145 cm^3^/min). The results demonstrate that the optimal parameters substantially improve machining stability and reduce mechanical loads, at the expense of productivity—a trade-off that is well justified in finishing operations where surface quality and machining precision are prioritized over material removal efficiency. The close agreement between predicted and measured values further validates the effectiveness of the proposed modeling and optimization framework. This framework enables workshops to adapt parameter selection to varying production requirements, improving both machining quality and operational efficiency.

## 4. Discussion

Most existing studies merely employ a single optimization algorithm or an independent decision-making method. In this study, orthogonal experimental modeling, multi-objective searching based on MOWOA, and objective decision-making via AHP-TOPSIS are integrated into a complete workflow, which can serve as a reference for multi-objective optimization of other cutting processes such as turning and milling.

Range analysis was carried out for the machine tool vibration acceleration and milling force. The results reveal that the descending order of influence degree of milling parameters on vibration acceleration is spindle speed *n* > axial milling depth *a*_p_ > feed rate *v*_f_. In terms of milling force, the influence ranking is axial milling depth *a*_p_ > feed rate *v*_f_ > spindle speed *n*.

The MOWOA algorithm is applied to conduct multi-objective optimization targeting vibration acceleration, milling force and material removal rate, and a set of Pareto-optimal solutions of optimized milling parameters is obtained. This work extends the application scenarios of MOWOA in the field of mechanical process optimization. Meanwhile, the combination of AHP and TOPSIS enriches the theoretical system of post-optimization decision-making for multi-objective problems. Comprehensive evaluation and ranking of all milling schemes are performed according to practical decision demands, and the optimal milling scheme satisfying user requirements is finally determined. The globally optimal process parameter set is determined as: spindle speed *n* = 12,000.00 r/min, feed rate *v*_f_ = 1048.26 mm/min, and axial milling depth *a*_p_ = 3.00 mm. Based on the weights assigned by the decision-maker, the optimized parameters achieve a 41.50% reduction in vibration acceleration and a 16.03% reduction in milling force compared with conventional machining parameters, accompanied by a 44.46% drop in material removal rate. The results demonstrate that substantial improvements in vibration suppression and milling-force mitigation can be obtained at the cost of partial machining efficiency, which is consistent with the decision-maker’s priority toward machining quality. From an industrial perspective, the optimized parameters offer a reliable solution for pursuing superior surface quality with low-vibration operation in high-precision machining sectors. For general-purpose machining workshops, the TOPSIS comprehensive score of milling experimental schemes provides a practical reference for parameter selection, enabling operators to make informed decisions according to specific production priorities. In terms of cost management, mitigated tool wear and improved surface quality help to lower overall production costs by reducing consumable consumption and minimizing rework operations, despite the longer processing time for each workpiece.

Despite the promising optimization results obtained in this work, several limitations should be noted. The multi-objective optimization framework is constructed based on three measurable responses: vibration acceleration, milling force, and material removal rate. Vibration and milling force are adopted as surrogate indicators that indirectly reflect surface quality and tool condition for wood engraving-milling, while MRR quantifies machining productivity. However, some critical machining outputs, including surface roughness, tool wear, and cutting temperature, are not incorporated into the optimization objectives. Quantitative characterization of cutting temperature, surface roughness and tool wear in wood-cutting experiments requires long-duration continuous cutting tests, which goes beyond the scope of the present orthogonal experimental campaign. Consequently, the obtained Pareto-optimal solutions cannot comprehensively capture the full practical machining behavior.

All experiments in this manuscript were carried out on a specific CNC engraving-milling machine, using one type of wood workpiece and a single cutting tool, within a predefined range of process parameters. Therefore, the established regression models, Pareto-optimal solutions, and derived optimal parameter combinations are only valid under the given experimental boundary conditions. Direct extrapolation to other wood species, different cutters, alternative machine-tool systems, or parameter ranges far beyond the experimental domain is not guaranteed. Even with these constraints, the proposed multi-objective optimization framework integrating orthogonal experiments, the predictive model, MOWOA algorithm and AHP-TOPSIS decision-making possesses general methodological reference value for wood-milling parameter optimization.

Future research can focus on improving the convergence strategy of the whale optimization algorithm tailored to milling optimization problems and developing hybrid intelligent optimization algorithms to further enhance the solution accuracy of Pareto fronts. In addition, multi-objective optimization models integrating surface roughness, tool wear and cutting temperature should be established to meet modern manufacturing requirements for low-carbon green machining and high-precision machining.

## Figures and Tables

**Table 1:** Tool performance parameters.

Model	Parameters ^1^				Density *ρ* (g/cm^3^)	Hardness *H*_R_ (HRA)	Bending Strength *σ*_b_ (MPa)
	*L* (mm)	*D* (mm)	*d* (mm)	*γ* (°)			
YG6C	100	8	2	30	14.8	88	1450

^1^ Parameters: *L* is the total tool length, *D* is the shank diameter, *d* is the cutting edge diameter, and *γ* is the tip angle.

**Table 2:** Workpiece performance parameters.

Workpiece	Dimensions *L × W × H* (mm)	Janka Hardness *HJ* (N)	Density *ρ* (g/cm^3^)	Elastic Modulus *E* (GPa)
Rosewood	120 × 80 × 8	2100	0.9	12

**Table 3:** Factors and levels for the experiment.

Level	Factors		
	*n* (r/min)	*v*_f_ (mm/min)	*a*_p_ (mm)
1	12,000	1000	3
2	15,000	1500	4
3	18,000	2000	5

**Table 4:** Random consistency index *RI*.

Order *m*	1	2	3	4	5	6
Value of *RI*	0	0	0.58	0.90	1.12	1.24

**Table 5:** Measured vibration acceleration *a* and milling force *F* in orthogonal milling tests.

NO	Factors			Results	
	*n* (r/min)	*v*_f_ (mm/min)	*a*_p_ (mm)	*a* (m/s^2^)	*F* (N)
1	12,000	1000	3	3.832	39.81
2	12,000	1500	4	4.818	50.13
3	12,000	2000	5	5.273	58.06
4	15,000	1000	3	4.356	39.46
5	15,000	1500	4	5.972	48.23
6	15,000	2000	5	7.38	56.56
7	18,000	1000	4	6.412	44.55
8	18,000	1500	5	7.805	60.83
9	18,000	2000	3	7.514	48.34
10	12,000	1000	5	5.208	45.23
11	12,000	1500	3	4.328	42.42
12	12,000	2000	4	4.625	55.05
13	15,000	1000	4	5.01	45.28
14	15,000	1500	5	7.181	52.43
15	15,000	2000	3	5.353	47.02
16	18,000	1000	5	7.806	54.82
17	18,000	1500	3	6.793	40.05
18	18,000	2000	4	8.632	52.85

**Table 6:** RA of vibration acceleration *a*.

Item for Analysis	*K_i_*	*n* (r/min)	*v*_f_ (mm/min)	*a*_p_ (mm)
*a* (m/s^2^)	*K*_1_	4.681	5.437	5.363
	*K*_2_	5.875	6.150	5.912
	*K*_3_	7.494	6.463	6.776
	Range	2.813	1.026	1.413

**Table 7:** ANOVA of vibration acceleration *a*.

Source	Sum Sq.	d.f.	Mean Sq.	*F*-Value	*p*-Value	Significance
*n*	23.9184	2	11.9592	44.19	0.002	**
*v*_f_	3.314	2	1.657	6.12	0.034	*
*a*_p_	6.0876	2	3.0438	11.25	0.0147	*
Error (pooled) ^a^	2.1647	8	0.2706	—	—	—
Total	35.4847	17	—	—	—	—

** Highly significant (*p*-value < 0.01); * significant (0.01 ≤ *p*-value < 0.05). ^a^ Error term is pooled from the four empty columns (e1–e4) of the L18 orthogonal array.

**Table 8:** RA of milling force *F*.

Item for Analysis	*K_i_*	*n* (r/min)	*v*_f_ (mm/min)	*a*_p_ (mm)
*F* (N)	*K*_1_	48.450	44.858	42.850
	*K*_2_	48.163	49.015	49.348
	*K*_3_	50.240	52.980	54.655
	Range	2.077	8.122	11.805

**Table 9:** ANOVA of milling force *F*.

Source	Sum Sq.	d.f.	Mean Sq.	*F*-Value	*p*-Value	Significance
*n*	15.198	2	7.599	1.37	0.305	n.s.
*v*_f_	197.921	2	98.961	17.79	0.0010	**
*a*_p_	419.494	2	209.747	37.71	6.98 × 10^−5^	**
Error (pooled) ^a^	44.489	8	5.561	—	—	—
Total	677.102	17	—	—	—	—

^a^ Error term is pooled from the four empty columns (e1–e4) of the L18 orthogonal array; n.s., not significant; ** Highly significant (*p*-value < 0.01).

**Table 10:** Significance of interaction and quadratic terms in the quadratic response surface model.

Term	Coefficient	Std. Error	t-stat	*p*-Value	Significance
*n*∙*v*_f_	−5.301 × 10^−7^	6.051 × 10^−7^	−0.876	0.407	n.s.
*n*∙*a*_p_	8.395 × 10^−4^	3.026 × 10^−4^	2.775	0.024	*
*v*_f_∙*a*_p_	−2.763 × 10^−4^	1.815 × 10^−3^	−0.152	0.883	n.s.
*n* ^2^	1.275 × 10^−7^	1.334 × 10^−7^	0.955	0.367	n.s.
*v*_f_^2^	2.135 × 10^−6^	4.803 × 10^−6^	0.445	0.668	n.s.
*a*_p_^2^	−0.397	1.201	−0.331	0.749	n.s.

* significant (0.01 ≤ *p*-value < 0.05); n.s., not significant.

**Table 11:** Regression coefficients and 95% confidence intervals of the power-law model.

Coefficient	Estimate	95% Confidence Interval
*C*	3.572 × 10^−6^	[3.5680 × 10^−6^, 3.5756 × 10^−6^]
*γ*_1_	1.207	[1.2000, 1.2132]
*γ*_2_	0.286	[0.28273, 0.29004]
*γ*_3_	0.469	[0.46407, 0.47395]

**Table 12:** Predictive performance of the power-law model.

Performance Metric	Calibration (Full Data)	Generalization (LOOCV)
RMSE	0.3626	0.4807
MAE	0.3317	0.4356
MAPE	5.79%	7.48%
***R*** **^2^**	0.934	—

**Table 13:** Regression coefficients and 95% confidence intervals of the quadratic response surface model.

Coefficient	Estimate	95% Confidence Interval
*β*_0_	70.821	[−3.1519 × 10^1^, 1.7316 × 10^2^]
*β*_1_	−6.100 × 10^−3^	[−1.6011 × 10^−2^, 3.8117 × 10^−3^]
*β*_2_	0.012	[−3.1710 × 10^−2^, 5.5775 × 10^−2^]
*β*_3_	−3.298	[−2.8009 × 10^1^, 2.1412 × 10^1^]
*β*_12_	−5.301 × 10^−7^	[−1.9255 × 10^−6^, 8.6527 × 10^−7^]
*β*_13_	8.395 × 10^−4^	[1.4181 × 10^−4^, 1.5372 × 10^−3^]
*β*_23_	−2.763 × 10^−4^	[−4.4625 × 10^−3^, 3.9099 × 10^−3^]
*β*_11_	1.275 × 10^−7^	[−1.8020 × 10^−7^, 4.3511 × 10^−7^]
*β*_22_	2.135 × 10^−6^	[−8.9404 × 10^−6^, 1.3211 × 10^−5^]
*β*_33_	−0.397	[−3.1659 × 10^0^, 2.3719 × 10^0^]

**Table 14:** Predictive performance of the quadratic response surface model.

Performance Metric	Calibration (Full Data)	Generalization (LOOCV)
RMSE	1.5721	3.7302
MAE	1.3388	3.1516
MAPE	2.77%	6.55%
*R* ^2^	0.939	—

**Table 15:** Statistical results of MOWOA over 30 independent runs.

Indicator	Mean	Standard Deviation	Coefficient of Variation
HV	0.0119	0.0001	1.15%
Number of Pareto solutions	150	0	0

**Table 16:** Sensitivity analysis results under representative weight configurations.

*ω*_a_	*ω*_F_	*ω*_Q_	Optimal Solution	*a* (m/s^2^)	*F* (N)	*Q* (cm^3^/min)	Trend
0.3	0.233	0.467	Solution E	5.6027	57.0233	10	Productivity-oriented
0.42	0.193	0.387	Solution E	5.6027	57.0233	10	Productivity-oriented
0.54	0.153	0.307	Solution D	5.3288	55.3976	8.913	Transition region
0.571	0.143	0.286	Solution A	3.6653	40.159	3.1448	Quality-oriented
0.68	0.107	0.213	Solution B	4.2175	47.2659	5.1369	Quality-oriented
0.8	0.067	0.133	Solution C	3.8607	42.191	3.7712	Quality-oriented

**Table 17:** TOPSIS comprehensive score of milling experimental schemes.

No	Parameters			Evaluation Indices			TOPSIS Score
	*n* (r/min)	*v*_f_ (mm/min)	*a*_p_ (mm)	*a* (m/s^2^)	*F* (N)	*Q* (cm^3^/min)	
1	12,000.0000	1048.2609	3.0000	3.6653	40.1590	3.1448	0.009560944
2	12,000.0000	1147.3818	3.0000	3.7612	41.1004	3.4421	0.009559898
3	12,000.0000	1173.2089	3.0000	3.7852	41.3525	3.5196	0.009556657
4	12,000.0000	1000.0000	3.0000	3.6162	39.7159	3.0000	0.009555547
5	12,000.0000	1257.0750	3.0000	3.8607	42.1910	3.7712	0.009536426
6	12,000.0000	1287.4709	3.0000	3.8872	42.5023	3.8624	0.009525192
7	12,223.3090	1000.0000	3.0000	3.6976	39.4888	3.0000	0.009517918
8	12,000.0000	1343.2703	3.0000	3.9347	43.0840	4.0298	0.009498788
9	12,000.0000	1400.7779	3.0000	3.9821	43.6975	4.2023	0.009463441
10	12,000.0000	1428.7879	3.0000	4.0047	44.0014	4.2864	0.009443106
11	12,000.0000	1485.4469	3.0000	4.0495	44.6264	4.4563	0.009395391
12	12,000.0000	1564.1785	3.0000	4.1098	45.5176	4.6925	0.00931451
13	12,000.0000	1580.9409	3.0000	4.1223	45.7108	4.7428	0.009295162
14	12,000.0000	1255.1828	3.5430	4.1723	44.2522	4.4472	0.009286266
15	12,973.2765	1177.9973	3.0000	4.1637	40.4111	3.5340	0.009264608
16	12,000.0000	1712.2886	3.0000	4.2175	47.2659	5.1369	0.009117043
17	13,345.2928	1111.6132	3.0907	4.2969	39.9705	3.4356	0.009093755
18	12,888.6895	1472.0523	3.0000	4.4028	43.4267	4.4162	0.009040622
19	12,000.0000	1460.0914	3.5870	4.3819	46.5503	5.2373	0.009008221
20	12,000.0000	1653.8344	3.2885	4.3596	47.6674	5.4387	0.008960686
21	13,053.3065	1396.1839	3.1028	4.4738	42.9659	4.3321	0.008945751
22	14,161.5331	1090.6425	3.0000	4.5274	38.7893	3.2719	0.008793224
23	12,000.0000	1729.0045	3.3542	4.4565	48.8062	5.7994	0.008763115
24	14,533.9651	1000.0000	3.0000	4.5570	37.8867	3.0000	0.008705417
25	14,280.3581	1134.2954	3.0000	4.6249	39.0913	3.4029	0.008678254
26	13,969.0856	1228.3608	3.1288	4.6990	40.8272	3.8433	0.008591983
27	14,773.9476	1012.1553	3.0000	4.6641	37.8913	3.0365	0.00856482
28	12,000.0000	1746.9837	3.5883	4.6134	49.8522	6.2686	0.008483026
29	14,752.1162	1096.1713	3.0000	4.7631	38.5585	3.2885	0.008459113
30	12,000.0000	1556.9091	4.0723	4.7368	49.2275	6.3402	0.008367916

**Table 18:** Comparison of machining performance between conventional and optimal parameter settings.

No	*a* (m/s^2^)			*F* (N)			*Q* (cm^3^/min)
	Predicted Values	Measured Values	Error %	Predicted Values	Measured Values	Error %	
1 ^a^	3.665	3.467	5.71	40.159	38.642	3.93	3.145
2 ^b^	6.0839	6.421	5.25	48.004	49.543	3.11	6.000

^a^ Optimal parameter settings: *n* = 12,000.00, *v*_f_ = 1048.26 mm/min, *a*_p_ = 3.00 mm. ^b^ Conventional parameter settings: *n* = 15,000.00, *v*_f_ = 1500.00 mm/min, *a*_p_ = 4.00 mm.

## Data Availability

The original contributions presented in this study are fully included within the manuscript. Further inquiries can be directed to the corresponding author.

## References

[1] Bilgin G. (2026). Performance Analysis of Advanced Feature Extraction Methods for Manufacturing Defect Detection via Vibration Sensors in CNC Milling Machines. Sensors.

[2] Li Q., Zeng P., Wu Q., Zhang Z. (2025). RAPSO: An Integrated PSO with Reinforcement Learning and an Adaptive Weight Strategy for the High-Precision Milling of Elastic Materials. Sensors.

[3] Li C., Zhao G., Meng F. (2024). Multi-objective optimization of machining parameters in complete peripheral milling process with variable curvature workpieces. J. Manuf. Process..

[4] Xu T., Huo Z., Wang T. (2023). Multi-objective optimization of gas-steam-power system for an integrated iron and steel mill considering carbon emission reduction and cost. J. Clean. Prod..

[5] Yang A., Jiang Q., Lin Y. (2026). A Two-Stage Fitness Learning Model-Driven Evolutionary Algorithm for Imbalanced Multimodal Multi-Objective Optimization. Symmetry.

[6] Zhang Y., Wang C., Bai Q., Zhang Q., He X. (2026). Multi-Objective Process Optimization of Micro-Milling Titanium Alloy Ti6Al4V for Microgrooves. Materials.

[7] Cica D., Tesic S., Markovic M., Sredanovic B., Borojevic S., Zeljkovic M., Kramar D., Pušavec F. (2025). Multi-Objective Optimization of Milling Ti-6Al-4V Alloy for Improved Surface Integrity and Sustainability Performance. Machines.

[8] Khan M.A., Jaffery S.H.I., Khan M.A., Faraz M.I., Mufti S. (2023). Multi-Objective Optimization of Micro-Milling Titanium Alloy Ti-3Al-2.5V (Grade 9) Using Taguchi-Grey Relation Integrated Approach. Metals.

[9] Li Z., Chen J., Meng Y., Zhu J., Li J., Zhang Y., Li C. (2022). Multi-Objective Optimization of Sugarcane Milling System Operations Based on a Deep Data-Driven Model. Foods.

[10] Sheheryar M., Khan M.A., Jaffery S.H.I., Alruqi M., Khan R., Bashir M.N., Petru J. (2022). Multi-Objective Optimization of Process Parameters during Micro-Milling of Nickel-Based Alloy Inconel 718 Using Taguchi-Grey Relation Integrated Approach. Materials.

[11] Lahlali B., El Abdelaoui F.Z., Ech-chaouy H. (2026). Multi-objective optimization of CNC milling parameters for sustainable manufacturing: Experimental analysis of energy consumption and noise emission. Int. J. Adv. Manuf. Technol..

[12] Pan Z., Yao B., Cai Z. (2026). Optimizing milling parameters for Inconel 718 with C60 nanofluid lubrication. J. Braz. Soc. Mech. Sci. Eng..

[13] Natesh C.P., Siddeshkumar N.G., Srinivasa G., Shivaramakrishna A., Pruthvi H.M., Prasad C.D., Shashidhara Y.M., Amarendra H.J., Tiwari A., Aden A.A. (2025). Multi-objective optimization of surface roughness and MRR in AISI 316L stainless steel processed by MQL end milling using taguchi, RSM, ANN, and RFR methods. Sci. Rep..

[14] Devi C., Mahalingam S.K., Cep R., Elangovan M. (2024). Optimizing end milling parameters for custom 450 stainless steel using ant lion optimization and TOPSIS analysis. Front. Mech. Eng..

[15] Makhadmeh S.N., Kassaymeh S., Rjoub G., Bataineh B., Sanjalawe Y., Al-Betar M.A. (2025). Recent advances in multi-objective whale optimization algorithm, its versions and applications. J. King Saud. Univ. Comput Inf. Sci..

[16] Tu B., Bao J., Zhou H., Huo Y., Han X., Hui N. (2026). Solving the 3D UAV Path Planning Problem Using an Improved Multi-Leader Multi-Objective Whale Optimization Algorithm. Biomimetics.

[17] Yang Y., Wei X. (2025). Optimization of Process Parameters for Surface Roughness and Milling Power of AL7075 CNC Milling Based on a Hybrid Multi-Objective Particle Swarm Optimization Integrating Whale Optimization Algorithm. Integr. Mater. Manuf. Innov..

[18] Beltran M., Wang B., Tan Y., Choi J., Lee D., Su L. (2026). Mechanochemistry-Driven Optimization of Halide-Based Solid-State Electrolytes via Orthogonal Design of Experiments and Regression Modeling. ACS Mater. Lett..

[19] Wang X., Liu Q., Wang H., Li X., Wang Q., Song G., Wang M. (2026). Machining behavior and milling force modeling of powder metallurgy Superalloy under nanofluid minimum quantity lubrication. Int. J. Adv. Manuf. Technol..

[20] Zhang C., Wang J., Jiao F., Cao Y. (2024). Research on tool wear and surface quality in laser-assisted ultrasonic elliptical vibration cutting of cemented carbide. Tribol. Int..

[21] Zhou J., Shi J., Zhao Y., Liu P., Meng X., Chen H. (2026). Cutting Performance of YG8 Cemented Carbide Tools with Microcapsule-Filled Surface Microtextures. Materials.

[22] Bembenek M., Kudelski R., Pawlik J., Kowalski Ł. (2021). The Influence of CNC Turning with VBMT, RCMX, 3ER, and MGMN Type Indexable Inserts on West African Ebony/*Diospyros crassiflora*, San Domingo Boxwood/*Phyllostylon brasiliense*, Rio Rosewood/*Dalbergia nigra*, Beechwood/*Fagus sylvatica*, Oakwood/*Quercus robur*, and Pinewood/*Pinus silvestris* Surface Roughness. Materials.

[23] Pinkowski G., Piernik M., Wołpiuk M., Krauss A. (2024). Effect of Chip Thickness and Tool Wear on Surface Roughness and Cutting Power during Up-Milling Wood of Different Density. BioResources.

[24] Gopal M., Gutema E.M., Lemu H.G., Sori J. (2024). A Hybrid Nondominant-Based Genetic Algorithm (NSGA-II) for Multiobjective Optimization to Minimize Vibration Amplitude in the End Milling Process. Adv. Mater. Sci. Eng..

[25] Lin Y., Ma J., Lai D., Zhang J., Li W., Li S., He S. (2022). Multi-response optimization of process parameters in nitrogen-containing gray cast iron milling process based on application of non-dominated ranking genetic algorithm. Heliyon.

[26] Chen H., An Y.-C. (2024). Green Residential Building Design Scheme Optimization Based on the Orthogonal Experiment EWM-TOPSIS. Buildings.

[27] Jenarthanan M.P., Prakash A.L., Jeyapaul R. (2016). Mathematical modeling of delamination factor on end milling of hybrid GFRP composites through RSM. Pigment Resin Technol..

[28] Benchiheub S., Bacha M.F. (2025). Multi-objective optimisation of milling parameters for Z200C12 and Z30C13 steels using ANOVA and desirability functions. Int. J. Adv. Manuf. Technol..

[29] Hernández L.W., Seid Ahmed Y., Curra D.A., Pérez R. (2025). Multi-Response Optimization of Milling Parameters of AISI D2 Steel Using Response Surface Methodology and Desirability Function. J. Manuf. Mater. Process..

[30] Deng C., Feng Y., Miao J., Ma Y., Wei B. (2019). Multi-Objective Machining Parameters Optimization for Chatter-Free Milling Process Considering Material Removal Rate and Surface Location Error. IEEE Access.

